# Biochar from Wood Chips and Corn Cobs for Adsorption of Thioflavin T and Erythrosine B

**DOI:** 10.3390/ma15041492

**Published:** 2022-02-17

**Authors:** Martin Pipíška, Eva Klára Krajčíková, Milan Hvostik, Vladimír Frišták, Libor Ďuriška, Ivona Černičková, Mária Kaňuchová, Pellegrino Conte, Gerhard Soja

**Affiliations:** 1 Department of Chemistry, Faculty of Education, Trnava University in Trnava, Priemyselná 4, P.O. Box 9, SK-918 43 Trnava, Slovakia; krajcikova.evaklara@gmail.com (E.K.K.); milan.hvostik@gmail.com (M.H.); vladimir.fristak@truni.sk (V.F.); 2 Institute of Materials Science, Faculty of Materials Science and Technology in Trnava, Slovak University of Technology in Bratislava, J. Bottu 25, SK-917 24 Trnava, Slovakia; libor.duriska@stuba.sk (L.Ď.); ivona.cernickova@stuba.sk (I.Č.); 3 Institute of Earth Resources, Faculty of Mining, Ecology, Process Control and Geotechnologies, Technical University of Košice, Park Komenského 19, SK-042 00 Košice, Slovakia; maria.kanuchova@tuke.sk; 4 Department of Agricultural, Food and Forestry Science, University of Palermo, 90128 Palermo, Italy; pellegrino.conte@unipa.it; 5 Energy Department, AIT Austrian Institute of Technology GmbH, Konrad-Lorenz-Straße 24, 3430 Tulln an der Donau, Austria; gerhard.soja@ait.ac.at; 6 Institute for Chemical and Energy Engineering, University of Natural Resources and Life Sciences, Muthgasse 107, 1190 Vienna, Austria

**Keywords:** wood chips, corn cobs, biochar, thioflavin T, eryhrosine B, adsorption kinetics, isotherm

## Abstract

Biochars from wood chips (WC) and corn cobs (CC) were prepared by slow pyrolysis and used for sorption separation of erythrosine B (EB) and thioflavin T (TT) in batch experiments. Biochar-based adsorbents were extensively characterized using FTIR, XRD, SEM-EDX, and XPS techniques. The kinetics studies revealed that adsorption on external surfaces was the rate-limiting step for the removal of TT on both WC and CC biochar, while intraparticle diffusion was the rate-limiting step for the adsorption of EB. Maximal experimental adsorption capacities *Q_maxexp_* of TT reached 182 ± 5 (WC) and 45 ± 2 mg g^−1^ (CC), and EB 12.7 ± 0.9 (WC) and 1.5 ± 0.4 mg g^−1^ (CC), respectively, thereby indicating a higher affinity of biochars for TT. The adsorption mechanism was found to be associated with π-π interaction, hydrogen bonding, and pore filling. Application of the innovative dynamic approach based on fast-field-cycling NMR relaxometry indicates that variations in the retention of water-soluble dyes could be explained by distinct water dynamics in the porous structures of WC and CC. The obtained results suggest that studied biochars will be more effective in adsorbing of cationic than anionic dyes from contaminated effluents.

## 1. Introduction

In recent decades, there has been an enormous raise in the production and application of synthetic dyes. The global dye and pigment market size was estimated at USD 32.9 billion in 2020, and from 2021 to 2028 is projected to rise at a compound annual growth rate of 5.1% as expected by Grand View Research [1]. Therefore, it is not surprising that the long-term utilization of synthetic dyes in a different range of industries such as the textile and food industries and the manufacture of pulp, paper, and dyestuffs have resulted in widespread water contamination [2,3]. Due to their low fixation rates, a large number of dyes are discharged into wastewater, and although currently used dyes (e.g., in the textile industry) are generally stable and not highly toxic or carcinogenic, in some circumstances their degradation products could be more harmful to the aquatic ecosystem [4]. In addition, many synthetic dyes are complexed with heavy metals (Ni, Cu, Cr), and during degradation processes these heavy metals are released, causing subsequent contamination of environment with the risk of entering the food chain [5].

Non-specific treatment processes for dye-containing effluents are required because a wide range of different dyes are used in the dyeing of textiles over a short period of time, and the effluents are therefore highly variable in composition [6,7]. Despite several successful processes using different physicochemical and biological principles, cost-effective removal of dyes from effluents continues to be a major challenge [8,9]. Adsorption is becoming an efficient and promising process among existing pollution-control technologies for the removal of synthetic dyes by various adsorbents of both organic and inorganic origin. The continuously growing number of research papers on dye adsorption suggests that there is still interest in improving conventional adsorption to an environmentally friendly approach. Recently, comprehensive reviews have been published summarizing current advances in the application of adsorption processes and outlining trends for the effective removal of synthetic dyes in the future [10,11].

Thioflavin T (TT, CI 49005) is a member of the cationic methine dye class, the most prominent group of methine dyes used extensively in the textile industry for the dyeing of polyacrylonitrile fibres [12]. Erythrosine B (EB, CI 45430) belongs to the category of water-soluble anionic xanthene dyes and is very commonly used, especially in the food, cosmetic, and pharmaceutical industries [13]. From these sources, dyes are introduced into water streams and thus have undesirable effects on public health [14]. Due to their potential toxicity and carcinogenicity, dyes are the subject of several environmental studies aimed at their removal from wastewater by adsorption processes [15,16,17,18].

Carbonaceous materials, especially the most commonly used activated carbon, have long been used to remove synthetic dyes due to their high specific surface area and porosity [19]. However, higher production costs limit their industrial application on a large scale. Lately, biochars have become the major research topic for many scientific groups worldwide [20] and have been increasingly studied and used as soil amendments [21,22,23] and for carbon sequestration in soils [24,25]. Due to their adsorption properties (high carbon content, porosity, and surface area) biochars represent a promising sustainable material for the separation of contaminants from contaminated environments [20,26,27]. According to a meta-analysis published by Alhashimi and Aktas [28], biochar as an adsorbent was found to have lower energy demand (6.1 MJ kg^−1^ vs. 97 MJ kg^−1^), lower global warming potential impact (−0.9 kg CO_2_eq kg^−1^ vs. 6.6 kg CO_2_eq kg^−1^), and lower environmental impacts than activated carbon. The authors further revealed that the adsorption capacity of biochar for heavy metals can compete with or exceed that of activated carbons. However, in the case of sorption separation of both anionic and cationic synthetic dyes, the adsorption capacities of biochars are extremely variable and vary from tens [29,30] to thousands [31,32] of mg g^−1^ depending on the feedstock, the type and conditions of pyrolysis, parameters of the adsorption process, dye properties, and removal mechanism. Further research on the study of biochar characterization and adsorption performance for synthetic dyes is of great importance to ensure the applicability of sorption-separation technology for industrial effluents. Moreover, there is no study in the literature regarding the adsorption of EB and TT by biochar-based adsorbents. In this study, we focused on the thermochemical conversion of waste wood chips and corn-cob biomass to biochars with the aim of preparing efficient adsorbent materials for synthetic dyes. The prepared biochars were accurately characterized (FTIR, XRD, XPS, SEM-EDX, FFC NMR relaxometry), utilized for adsorption of both anionic (erythrosine B) and cationic (thioflavin T) dyes from aqueous solutions, and kinetic and equilibrium phenomena was systematically investigated by means of batch adsorption experiments. 

## 2. Materials and Methods

### 2.1. Biochar Preparation

Biochar was produced from wood chips and corn cobs. The biomass was pyrolyzed in a customized laboratory pyrolysis reactor Arnold (AIT Vienna, Austria) employing a slow pyrolysis procedure at a maximum processing temperature of 500 °C. The residence time at maximum temperature was 2 h. Nitrogen (N_2_) as a flushing gas was used to ensure inert and uniform heating conditions. Both WC and CC biochars were washed twice thoroughly in Milli-Q-grade water (<0.4 μS cm^−1^, Simplicity 185, Millipore), oven-dried for 72 h at 90 °C to a constant mass, milled, and sieved; in all adsorption experiments the 0.5–1 mm fraction was used.

### 2.2. Biochar Characterization

The total C, H, N, and S contents of WC and CC biochars were quantified with a CHNS-O EA 1108 elemental analyzer (Carlo Erba Instruments, Milano, Italy). Oxygen content was calculated according to EBC guidelines. The pH values were determined after the biochar sample mixing with Milli-Q-grade water (1:2.5 ratio) for 1 h and stabilization for 1 h. The specific surface area (SSA) and pore properties of the prepared biochars were analyzed by the Brunauer–Emmett–Teller (BET) method with N_2_ as sorbate gas (Quantachrome Nova analyzer, Boynton Beach, FL, USA). The drift method was used to measure the pH of WC and CC biochars at the point of zero charge (pH_pzc_). The pH value for 25 mL of 0.1 M KNO_3_ solutions was adjusted in the range of 2.0 to 11.0 and 0.015 g of biochar was added. Sealed flasks were placed on a rotary mixer and shaken for 24 h at 45 rpm. Subsequently, the final pH was then measured and pH_pzc_ was determined from the plot (pH_in_ against pH_fin_). Infrared absorption spectra of both WC and CC biochar were measured by FT-IR (PerkinElmer Spectrum System 2000, Waltham, MA, USA) to characterize surface functional moieties. The samples were prepared in KBr with a ratio 1:200 (biochar:KBr) and pressed into pellets. FTIR spectra were obtained in the range 4000–400 cm^−1^. The morphology and microstructure observations of the BC surface structure and EDX microanalysis were performed using a JEOL JSM7600F scanning electron microscope (Tokyo, Japan) at 20 kV in the regime of secondary electrons. Before SEM and EDX analysis the BC samples were oven-dried at 80 °C for 24 h. Surface chemical analysis of BC was performed by X-ray photoelectron spectroscopy (XPS) with a SPECS XPS instrument (SPECS GmbH, Berlin, Germany) equipped with a PHOIBOS 100 SCD and a non-monochromatic X-ray source. Spectra were acquired at a base pressure of 2 × 10^−8^ mbar with AlK_α_ excitation at 10 kV (200 W) during the measurements. The survey surface spectrum was measured at transition energy of 70 eV and the core spectrum at 30 eV at room temperature. The data were analyzed using SpecsLab2 CasaXPS software. The energy scale was calibrated by normalizing the C1s carbon line to 284.5 eV. Curve fitting of the O1s spectra was performed using Gaussian–Lorentzian peak shaping after performing Shirley background correction.

X-ray diffraction (XRD) analysis was conducted using a Philips PW 1830 diffractometer (Philips N.V., Amsterdam, The Netherlands) with iron-filtered Co Kα1,2 radiation to determine the crystalline structure of the biochar employed in the adsorption experiments. The XRD patterns were acquired in the 2θ range between 10 and 80° with a step size of 0.02° and an exposure time of 10 s per step.

For fast-field-cycling (FFC) NMR relaxometry investigations, 1 g of dried and 2 mm sieved biochar was weighted in NMR tubes and 3 g of Milli-Q-grade water (electrical resistivity 18.2 MΩ cm at 25 °C) was added, followed by gentle stirring. The samples were put in the probe of Stelar Smartracer Fast-Field-Cycling Relaxometer (Stelar s.r.l., Mede, PV–Italy) and analyzed at a constant temperature of 25 °C. The basic theory about FFC NMR relaxometry has been already summarized by Conte (2019) [33]. Briefly, the proton spins were polarized at a polarization field (*B*_POL_) corresponding to a proton Larmor frequency (ω_L_) of 8 MHz for a period of polarization (*T*_POL_) corresponding to about five times the *T*_1_ estimated at this frequency. After each *B*_POL_ application, the magnetic field intensity (indicated as *B*_RLX_) was systematically changed in the proton Larmor frequency ω_L_ comprised in the range 0.01–10 MHz. The period τ, during which *B*_RLX_ was applied, was varied on 16 logarithmically spaced time sets, each of them adjusted at every relaxation field to optimize the sampling of the decay/recovery curves. Free induction decays (FID) were recorded following a single ^1^H 90° pulse applied at an acquisition field (*B*_ACQ_) corresponding to the proton Larmor frequency of 7.20 MHz. A time domain of 100 µs sampled with 1000 points was applied. Field-switching time was 3 ms, while spectrometer dead time was 15 µs. For all the experiments, a recycle delay of 6 s was used. A non-polarized FFC sequence was applied when the relaxation magnetic fields were in the range of the proton Larmor frequencies comprised between 10.0 and 3.6 MHz, whereas a polarized FFC sequence was applied in the proton Larmor frequencies *B*_RLX_ range of 3.6–0.01 MHz [34]. 

### 2.3. FFC NMR Data Elaboration

The longitudinal relaxation time (*T*_1_) values of the observed nuclei were obtained for each *B*_RLX_ by changing the *τ* values, as reported above. The relationship between signal intensity and *τ* is modeled by Equation (1):

(1) $$I\left(\tau \right)=I_{0}\exp{\left[\left(-\tau /T_{1}\right)\right]}^{k}$$

*I*_0_ is the ^1^H signal intensity at the thermal equilibrium, *I*(*τ*) is the ^1^H signal intensity at each fixed B_RLX_, *k* is a heterogeneity parameter related to the stretching of the decay process, and *T*_1_ is the average proton spin lattice relaxation time. The above function can be assumed to be a superposition of exponential contributions, and hence describes the likely physical picture of a particular distribution in *T*_1_.

Relaxation data at the proton Larmor frequency of 10 kHz were also evaluated by the inverse Laplace transformation through the application of the UPEN algorithm [33] to obtain the *T*_1_ distributions at such magnetic field and, therefore, information on pore distribution and water interaction. The resulting *T*_1_ distributions were fitted with a Gaussian function to obtain the deconvolution curves. 

### 2.4. Adsorption Experiments

Both erythrosine B (Acid Red 51, EB) with a reported purity of 95% and thioflavin T (Basic Yellow 1, TT) with a purity of 65% were obtained from Sigma-Aldrich (St. Louis, MO, USA). Selected physicochemical properties and molecular structures of EB and TT are presented in Table 1. Working solutions of EB and TT were prepared using deionized water taking into consideration the purity of the dyes. 

Adsorption kinetic tests were carried out by adding WC or CC biochar (suspension density 8.3 g L^−1^) to solutions of thioflavin T (100 mg L^−1^, pH 6.0) and erythrosin B (43 mg L^−1^, pH 6.0). The initial concentrations were selected on the basis of preliminary experiments. All adsorption experiments were performed with plastic vials using a batch procedure in a rotary mixer (45 rpm, 22 °C). At time intervals (30–2880 min), the tubes were centrifuged (8 min, 5500 rpm) and the absorbance in the supernatant after appropriate dilution was measured at λ_max_ = 526 nm (EB) or λ_max_ = 413 nm (TT) using a UV-VIS spectrophotometer (Cary 50, Varian, Mulgrave, Australia), and the concentrations of both EB and TT in the solutions were calculated from the calibration curves. The amounts of EB and TT *Q_eq_* (mg g^−1^) adsorbed by the biochars were calculated as follows:

(2) $$Q_{t}=\left(C_{0}-C_{t}\right)*V/M$$

where *C*_0_ is the initial and *C_t_* is the solution concentration of dye molecules at time *t* (mg L^−1^), *V* is total solution volume (L), and *M* represents biochar dosage (g).

The adsorption capacity of the tested biochars was examined in solutions with initial erythrosine B concentrations ranging from 13 to 309 mg L^−1^ and thioflavin T concentrations between 25 and 400 mg L^−1^. The vessels were shaken on a rotary mixer (45 rpm) at 22 °C and at the end of the experiments. The absorbance of the fluids was measured after separation of the biochar. Calculations of adsorption capacities *Q_eq_* (mg g^−1^) were made using the following equation:

(3) $$Q_{eq}=\left(C_{0}-C_{eq}\right)*V/M$$

where *C*_0_ is the initial and *C_eq_* equilibrium dye concentrations (mg L^−1^), *V* is the total solution volume (L), and *M* represents biochar dosage (g).

To characterize the influence of pH on dye adsorption, tested biochars were shaken in EB (*C*_0_ = 43 mg L^−1^) and TT solutions (*C*_0_ = 100 mg L^−1^) of desired initial pH (2.0–8.0) adjusted by adding 0.1 M NaOH or 0.1 M HCl. All adsorption experiments were performed in duplicate. 

## 3. Results and Discussion

### 3.1. Properties of the WC and CC Biochars

The principal physicochemical properties of the WC and CC biochar produced by the slow pyrolysis process are summarized in Table 2. The C, H, N, and S contents of WC were 79.9, 1.59, 0.45, and 0.18%, and of CC 82.8, 2.08, 1.39, and 0.19%. The WC and CC biochars were characterized by high carbon content and low H/C (0.24 and 0.31) and O/C (0.111 and 0.087) atomic ratios due to dehydration and decarboxylation reactions of cellulose, hemicelluloses, and lignin occurring at 500 °C associated with increasing aromaticity and the degree of condensation [36]. Moreover, lower H/C ratios indicate higher stability of biochars compared to initial feedstock. Both biochars produced were, as expected, alkaline in nature (pH = 8.74 CC; pH = 8.58 CC) and showed a relatively higher conductivity due to their large ash content (6.61% CC; 5.65% WC, respectively). The alkaline pH of biochar is attributed to the inorganic constituents present in the raw material, particularly alkaline earth metals such as K, Ca, and Mg [37]. 

As indicated in Table 2, the BET specific surface area values obtained for the biochars were 10.38 m^2^ g^−1^ for CC and 53.68 m^2^ g^−1^ for WC. This is consistent with the data from Liu et al. [38], according to which the specific surface area of biochars produced at temperatures above 450 °C is typically greater than 10 m^2^ g^−1^. The total pore volume was 0.021 cm^3^ g^−1^ for CC and 0.045 cm^3^ g^−1^ for WC. In the examined biochar there was a clear variation in the amount of micro- and mesopores. In the case of CC, micropores represented only 0.00005 cm^3^ g^−1^, with most of the volume being due to mesopores (0.019 cm^3^ g^−1^). In contrast, WC was characterized by a uniform representation of micro- (0.021 cm^3^ g^−1^) and mesopores (0.021 cm^3^ g^−1^). The SSA and the porous structure of the biochar-based adsorbent are often regarded as the crucial factors that govern the ability of the material to adsorb chemical compounds, and thus affect the adsorbent performance in liquid-phase adsorption. Therefore, the type and temperature of pyrolysis should be thoroughly optimized to obtain biochar with the required adsorptive characteristics.

**Figure 1:**
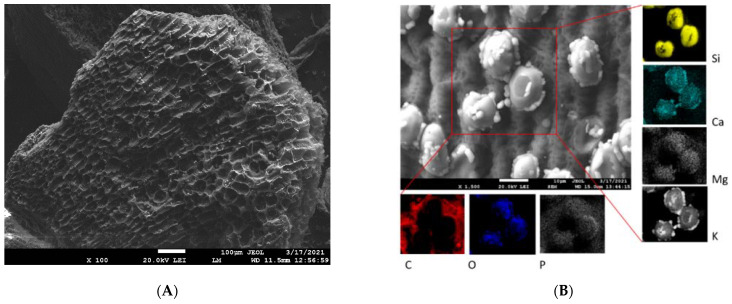
SEM (**A**) and EDX (**B**) images of CC biochar. EDX elemental distribution maps show elemental (C, O, P, Si, Ca, Mg, and K) composition of area closed by red lines in panel (**B**).

**Figure 2:**
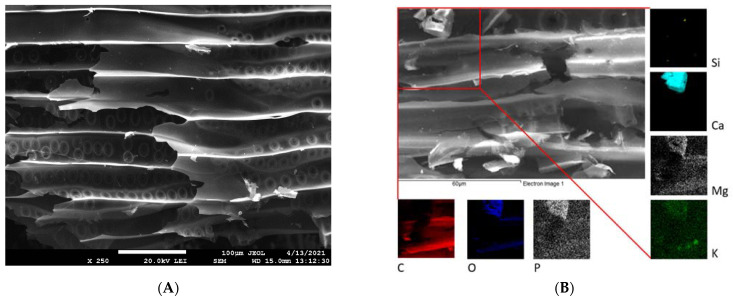
SEM (**A**) and EDX (**B**) images of WC biochar. EDX elemental distribution maps show elemental (C, O, P, Si, Ca, Mg, and K) composition of area closed by red lines in panel (**B**).

The example SEM images in Figure 1 and Figure 2 highlight the differences in surface structure and morphology between corn-cob and wood-chip biochars. The CC (Figure 1A,B) is represented by a honeycomb structure of macropores measuring 30–40 µm in diameter, separated by thin pore walls. Moreover, residues of glume elliptical protrusions are widely observed. Wood-chip biochar is characterized by the presence of tracheids and lateral communication between tracheids (bordered pits), and has slit-shaped pores from vesicles formed in the wood structure due to pyrolysis at 500 °C (Figure 2A,B). Detailed EDX elemental analysis confirmed the presence of K, Ca, Mg, P, and Si, in addition to the principal bulk elements of biochar (C, H, O). These mineral components are often present in biochar in the form of carbonates, phosphates, and oxides [39] and could significantly affect biochar remediation potential [25].

**Figure 3:**
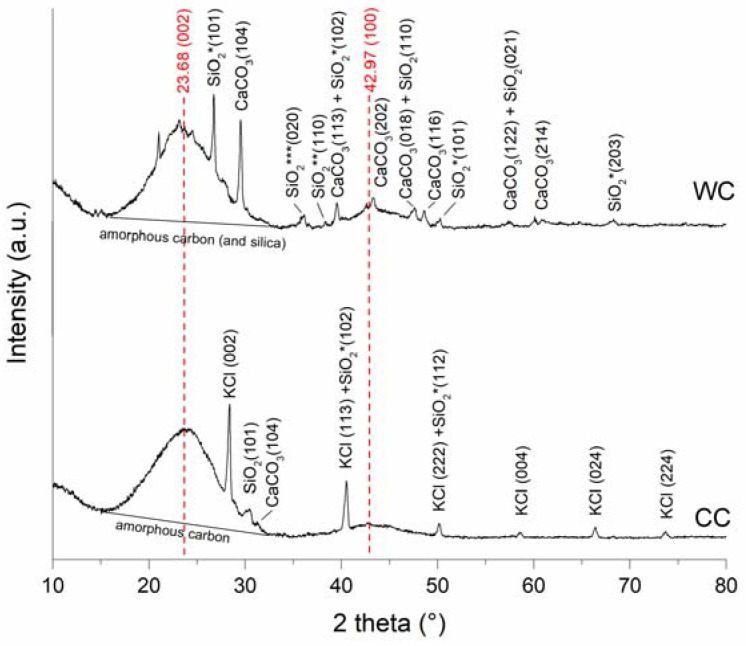
XRD diffraction pattern from wood-chip (WC) and corn-cob (CC) biochars.

XRD analysis was applied to determine the level of pyrolysis and identify the crystalline structure of biochars. The X-ray diffraction patterns are shown in Figure 3, and it is clear that both CC and WC biochar exhibited broad peaks at 2θ 23.68° (002) due to scattering of X-rays by amorphous carbon (and amorphous silica) [40], and 42.97° (100), indicating the presence of aromatic carbon and formation of turbostratic crystallites [41]. Generally, sharper C (002) and C (100) peaks indicate a higher degree of crystalline orientation of C in biochar samples [42]. Biochar produced from wood chips at 500 °C consisted of several other crystallite phases such as calcite (CaCO_3_) and quartz (SiO_2_). In contrast, corn-cob biochar was more strongly associated with the presence of sylvite (KCl) and quartz (SiO_2_), and only very slightly with calcite (CaCO_3_), and other mineral impurities. The higher carbonate content of WC biochar is probably related to the presence of calcium oxalate (CaO_x_) crystals in the leaves, wood, and bark of many plants, which transform to calcite at pyrolysis temperatures above 500 °C [43]. Moreover, the formation of calcite contributes to the alkalinity of the prepared CC and WC biochars, as shown by their relatively higher pH values (Table 2).

**Figure 4:**
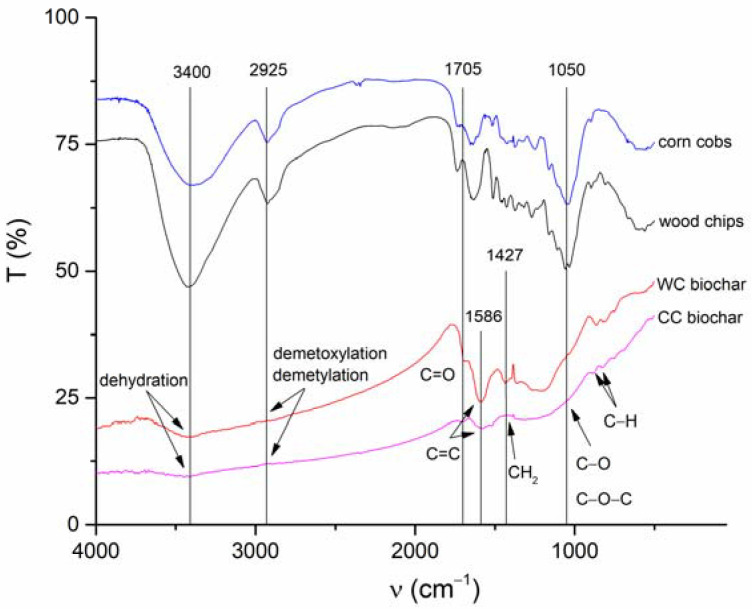
Fourier-transformed infrared bands characterizing surface functional moieties of both corn-cob and wood-chip feedstock and CC and WC biochars.

Surface functional moieties of BC and CC biochars and corresponding feedstock measured by FT-IR in the mid-infrared region are shown in Figure 4. It is clear that the FT-IR spectra of both biochars pyrolyzed at 500 °C compared to the feedstock material showed a substantial decrease in (i) O-H groups’ vibrations (3400 cm^−1^) as a result of dehydration, and (ii) aliphatic C-H stretch vibrations (2925 cm^−1^) because of lignin demethoxylation and demethylation during pyrolysis [20]. However, the aromatic ν(C=C) band at 1586 cm^−1^ and C-H bending aromatic CH out-of-plane deformation at 876, and 820 cm^−1^ have significant intensities in both studied biochars (Figure 4). The biochars’ low-intensity bands at 1050 and 1705 cm^−1^ are associated with carbonyl and carboxylic groups C-O and C=O stretching vibrations of undecomposed cellulosic and ligneous carbon [44]. The spectral properties of biochar are often related to the mineral fraction of BC. The abundance of carbonate (CO_3_^2−^) is suggested by peaks at 820 cm^−1^ and is in agreement with the presence of calcite in both the CC and WC biochars (Figure 3). In addition, the spectral region of 1140–1000 cm^−1^ matches the Si-O and Si-O-Si bonding, as indicated by EDX analysis (Si on the elemental map, Figure 1 and Figure 2) and XRD (Figure 3) analyses.

**Figure 5:**
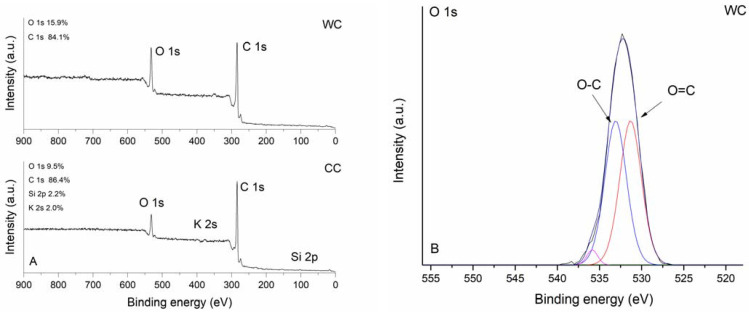

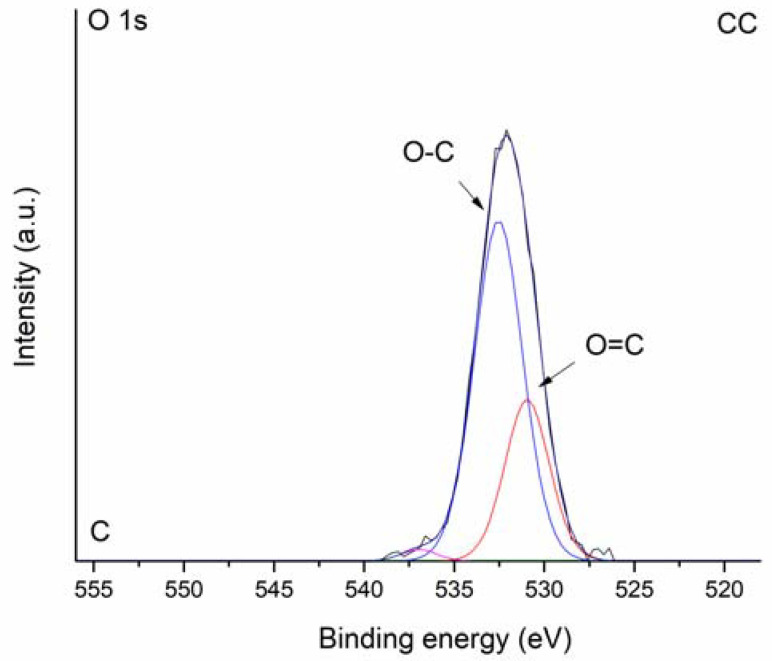
XPS survey spectra (**A**) and high-resolution O 1s spectra of WC (**B**) and CC (**C**) biochar.

The XPS survey spectra of studied biochars are shown in Figure 5A. As expected, characteristic strong carbon signals at 284.0 eV (C 1s) were observed for both CC and WC biochars together with varying amounts of oxygen detected at binding energy 531.5 or 532.5 eV (O 1s). The corn-cob biochar was notable for the relatively high presence of K (2.0 at%) and Si (2.2 at%). High-resolution XPS O 1s spectra were further analyzed to examine the nature of oxygen species on the surface of biochar samples. The O 1s band of CC and WC biochars (Figure 5B,C) were deconvoluted into the subsequent peaks: (i) peaks at 531.0 eV (CC) and 531.3 eV (WC) representing carbonyl oxygen; and (ii) peaks at 532.5 (CC) and 533.0 eV (WC) representing carbonyl oxygen in carboxylic and anhydrides, and O atoms in hydroxyl groups, which confirms the results found by FTIR. Moreover, it is evident that the intensity of the O-C component relative to the O=C component for CC is greater than for the WC biochar. Peaks at high binding energies 535.8 and 536.9 eV could be attributed to adsorbed water [45]. 

To evaluate the nature of biochar–water interactions, an innovative dynamic approach based on FFC NMR relaxometry was used [20] to measure the longitudinal relaxation times (*T*_1_). 

**Figure 6:**
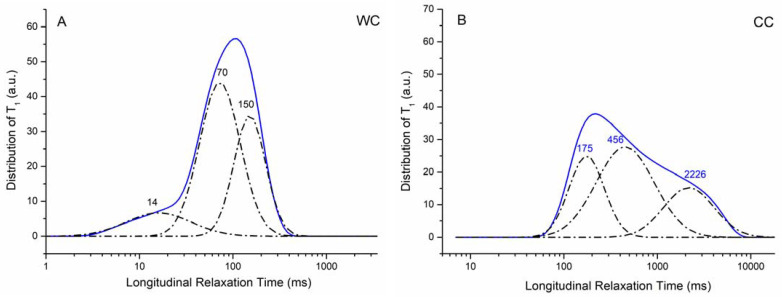
Relaxograms (i.e., distribution of longitudinal relaxation times) for the WC (**A**) and CC (**B**) biochar samples.

Proton longitudinal relaxation times (*T*_1_) in liquid systems within porous media are impacted by the collisions between the liquid-state molecules and the walls of the porous boundaries. Space restrictions (e.g., water in small-sized pores) do not allow fast molecular motions determining short relaxation times. If water strongly interacts with the porous surface or with polar functional groups on the solid surface, the proton dipolar interaction efficiency is enhanced and consequently, the *T*_1_ value shortens [46]. Conversely, an increase in water mobility (i.e., presence of large pores) weakens the dipolar interaction producing the shift in *T*_1_ values towards longer values. According to this, longitudinal relaxation times (*T*_1_) distributions at a fixed proton Larmor frequency (i.e., 10 kHz in our case) can be used to extrapolate the pore distribution of porous media [47]. Values of relaxation times are related to water mobility within the biochar’s porous surface. In the relaxograms, *T*_1_ values appear continuously distributed, representing the wide heterogeneity of the biochar’s porous system which contains different-sized pores (i.e., micro-, meso-, and macropores). Going from the left to the right hand of the two relaxograms in Figure 6, the *T*_1_ distribution indicates progressively unconstrained water molecules. In other words, the left part of the graph is produced by strongly bound water restricted in small-sized pores, whereas the right zone of the relaxogram is due to bulk water moving into large pores (e.g., macropores). The intermediate *T*_1_ values are generated by weakly bound water in mesopores [48]. 

The *T*_1_ values reported in Figure 6 are consistent with the values of the physical parameters reported in Table 2. In fact, the WC biochar showed a larger SSA value as compared to the CC biochar. Therefore, it is possible to hypothesize that water molecules are more constrained on the surface of the former than the latter system. Being less mobile, the ^1^H longitudinal relaxation time of the water molecules interacting with the WC biochar is shorter than that due to the molecules on the surface of the CC biochar. In addition, the amount of micropores in WC appears higher than in the CC biochar (see V_micro_ values in Table 2), thereby confirming that the liquid material is more constrained in WC than in CC.

The deconvolution of the relaxograms reported in Figure 6 (dot-dashed lines) provides three different Gaussian-shaped curves, each corresponding to water located in differently sized pores, all belonging to the micro- and meso-size intervals. In particular, the bands at 14 and 70 ms (Figure 6A) can be due to water moving in <2 nm pores [49], being the 70 ms band generated by water molecules inside the pores on the upper limit of the micro-pore interval. The bands at 150 (Figure 6A), 175, and 456 ms (Figure 6B) may be due to water moving in mesopores ranging from 2 to 50 nm [49]. Finally, the band centered at around 2226 ms can be attributed to the bulk water which usually shows a longitudinal relaxation time of 2.5 s [50].

### 3.2. Erythrosine B and Thioflavin T Adsorption

Prepared plant-based biochars were applied for the adsorption of synthetic organic dyes. In this work, we used the anionic dye erythrosine B (EB) and the cationic benzothiazole dye thioflavin T (TT) as model pollutants. EB belongs to the category of xanthene dyes and is used especially in the food industry. Due to its potential toxicity and carcinogenicity, it is the subject of several environmental studies aimed at its removal from wastewater [15,16].

**Figure 7:**
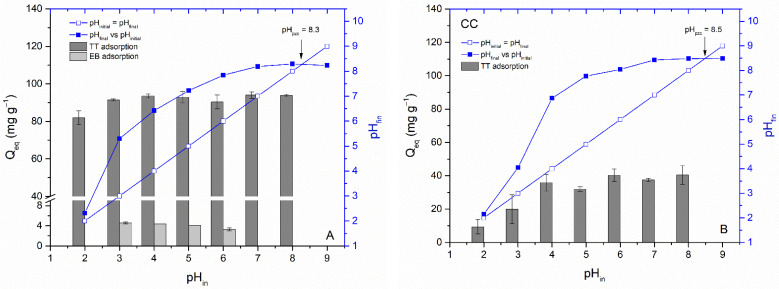
Determination of pH_pzc_ and the influence of pH_in_ on TT (100 mg L^−1^) adsorption by WC (**A**) and CC (**B**) and EB (45 mg L^−1^) adsorption by WC (**A**) biochar (8.3 g L^−1^; T = 22 °C).

*Effect of pH.* The solution’s pH has an impact on the ionization of both erythrosine-B and thioflavin-T molecules and the dissociation of oxygen-containing functional moieties (especially hydroxyl and carboxyl) on the biochar surfaces. The electrical state of the biochar surface is typically characterized by the point of zero charge, and the pH_pzc_ represents conditions in which the net surface charge on the biochar equals zero. The pH_pzc_ values of WC and CC biochars obtained by the pH drift method were 8.3 and 8.5, respectively (Figure 7A,B). This is consistent with previously published data indicating that biochar without further treatment has a pH_pzc_ greater than 6.0 [51,52]. This means that at solution pH lower than pH_pzc_, biochars will have a positive surface charge (due to the protonation of acidic groups), which could theoretically favor the adsorption of anionic dyes.

Figure 7A,B present the impact of initial pH on TT and EB adsorption by studied biochars. The adsorption capacity of WC for TT was only slightly affected at pH 2.0 and insignificantly affected by the solution pH from 3.0 to 8.0. More pronounced influence on TT adsorption at pH 2.0 and 3.0 were observed by CC biochar and could be attributed to electrostatic repulsion between positively charged CC biochar (pH_pzc_ = 8.5) and positively charged thioflavin T molecules. Despite the fact that the TT molecule has a positive charge (+1 e) on the N atom that is non-uniformly distributed between the molecule fragments and may contribute to some extent in retaining the TT on the biochar’s outer and inner surfaces at higher pH values, our data indicates that electrostatic attraction does not play a decisive role in the adsorption of TT by WC and CC biochars and other mechanisms/interactions such as pore filling, π-π interactions, and hydrogen bonding must be involved [53]. Interestingly, electrostatic attraction has an important role in TT adsorption by magnetically functionalized moss biosorbent due to the strong effect of the solution pH value on TT adsorption [12]. In the case of the anionic xanthene dye EB, we observed a slight increase in adsorption with a decrease in pH to 3.3 (Figure 7B). This may be due to an increase in electrostatic attraction between the positively charged WC biochar surfaces and the negatively charged EB (pKa 4.1). However, compared to TT, the adsorption capacity of EB is very low even at low pH values, indicating only limited applicability of WC and CC biochars for the removal of anionic dyes. 

**Figure 8:**
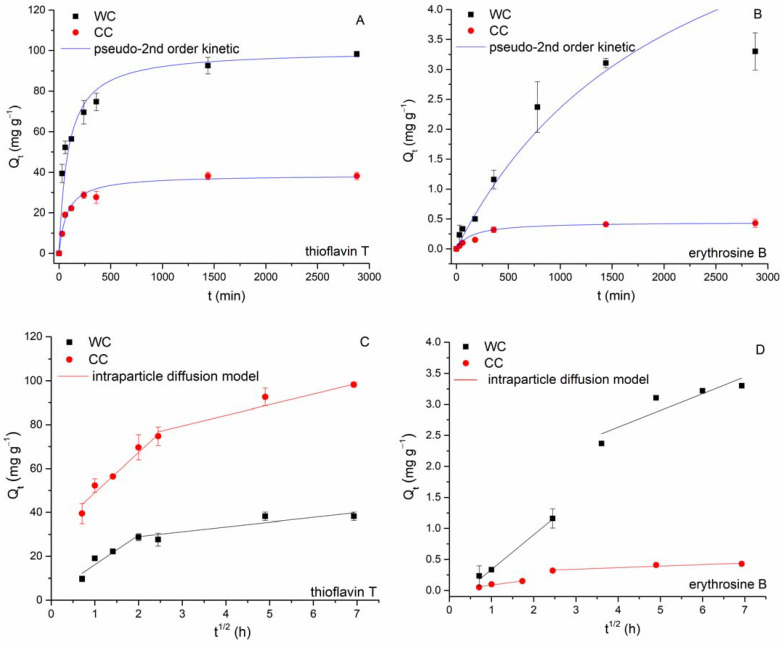
Effect of the reaction time on TT (C_0_ = 100 mg L^−1^, (**A**)) and EB (C_0_ = 43 mg L^−1^, (**B**)) adsorption by wood-chip and corn-cob biochars (8.3 g L^−1^; pH 6.0; T = 22 °C). Adsorption kinetics fitting by pseudo-second-order (**A**,**B**) and intraparticle-diffusion (**C**,**D**) models.

*Adsorption kinetics.* The time course of sorption of EB and TT is shown in Figure 8. It is evident that the biochars used differ significantly in their ability to adsorb both cationic (TT) and anionic (EB) dyes. 

The kinetics of TT sorption by both wood-chip and corn-cob biochars is typically a multiphase process. In the first phase (within the first 60 min), we observed rapid sorption due to a higher driving force, in which more than 50% of the total amount of adsorbed thioflavin T is captured (52.5 mg g^−1^ for WC; 19.0 mg g^−1^ for CC). The second phase proceeded more slowly until the sorption equilibrium was established after 24 h (CC) and 48 h (TT), respectively, and TT adsorption capacity reached 98.3 mg g^−1^ (WC) and 38.2 mg g^−1^ (CC), respectively. A different trend was found in EB adsorption. During the first phase (60 min), the adsorption capacity of both WC and CC reached only 10% of the total amount of adsorbed erythrosine B. The second phase is represented by a gradual increase in adsorption capacity (3.3 mg g^−1^ for WC; 0.43 mg g^−1^ for CC), and the equilibrium state was reached at 24 h. A similar kinetic profile for EB adsorption by pumpkin-seed hulls was observed by Apostol et al. [54] with equilibrium achieved after 3 h. 

As already mentioned in the literature, the sorption process of organic dyes [14] and other synthetic industrial chemicals [55] consists of several steps that take place simultaneously or successively. For porous adsorbents such as biochar, this includes external mass-transfer process, internal or intraparticle diffusion, and adsorbent–adsorbate surface interactions (e.g., chemisorption). To quantify both initial rapid and slow adsorption of EB and TT, kinetic data were analyzed by both reaction-controlled kinetics (pseudo-second-order kinetic model, PSO [56]) and diffusion-controlled kinetics (intraparticle diffusion model, IDM [57]) models, which can be described as follows:

(4) $$Q_{t}=(Q_{e}^{2\;}k_{2}^{\prime }t)/\left(1+k_{2}^{\prime }Q_{e}t\right)$$

(5) $$Q_{t}=k_{i}t^{0.5}+I$$

where *Q_t_* and *Q_e_* are the amount of EB and TT (mg g^−1^) adsorbed at time *t* (min) and equilibrium, respectively, *k_2_* is the pseudo-second-order rate constant (g/mg min), *I* is a constant that reflects the thickness of the boundary layer (mg g^−1^), and *k_i_* is the intraparticle diffusion rate constant (mg/g min^0.5^). 

It is apparent from Table 3 and Figure 8A,B that TT kinetics data for both CC and WC biochars were reasonably good, described with the PSO model with high values of *R*^2^ values and high comparability of *Q_eq cal_* to the experimental values of *Q_eq exp_*. However, the lesser suitability of the pseudo-second-order kinetic model to describe the kinetics of EB adsorption by WC is evident from Figure 8B. On the other hand, there is a multilinear relationship between *Q_t_* and *t*^0.5^ over a wide range of reaction times (Figure 8C,D), suggesting bulk diffusion and diffusion in water film surrounding biochar particles, followed by macro-/meso- and micro-pore diffusion [58,59]. For TT and EB adsorption by WC biochar, two separate regions were observed. During the first step, external surface adsorption or instantaneous adsorption of both dyes occurred, and 76% of TT (*k*_i1_ = 18.3) and 36% of EB (*k*_i1_ = 0.56) were rapidly bound by exterior biochar surfaces. The second linear region presented gradual adsorption where intraparticle diffusion was controlled and was characterized by the slow TT and EB diffusion rates (*k*_i2_ = 0.789 for EB and *k*_i2_ = 15.8 for TT) caused by slow solute movement from larger biochar pores to micropores [60]. Two separate regions were also observed when the CC biochar was applied for TT and EB adsorption (Figure 8C,D). During the first step, 79% of TT (*k*_i1_ = 13.7) and 35% of EB (*k*_i1_ = 0.09) were bound by exterior biochar surfaces. The second linear region was characterized by slow diffusion rates and an only a small portion of TT and EB was removed by CC biochar. This can be explained by both the smaller number of micropores in CC (Table 2) and the spatial limitation of the size of TT and EB molecules and pores. In addition, the *I* values of the WC biochar were apparently larger than those of the CC biochar, implying that the boundary layer in the WC was thicker than that in the CC. 

The observed differences in the kinetic profiles of the organic dyes are due to (i) different adsorption mechanisms of TT and EB (reaction-controlled vs. diffusion-controlled kinetics; cationic vs. anionic dye), (ii) different affinities of erythrosine B and thioflavin T for binding sites on the biochar surfaces, and (iii) different properties and structures of the biochars studied. Based on the results obtained, it can be assumed that TT adsorption on external surfaces was the rate-limiting step for the adsorption of thioflavin T on both WC and CC biochar, while intraparticle diffusion was the rate-limiting step for the adsorption of erythrosine B on WC.

*Adsorption equilibrium.* Isothermal tests were performed in batch mode (C_0_ TT 25–400 mg L^−1^; C_0_ EB 13–309 mg L^−1^, biochar dosage = 8.3 g L^−1^; pH 6.0; T = 22 °C) to determine the removal capacities of erythrosine B and thioflavin T using the WC and CC biochar. Freundlich (6) [61] and Langmuir (7) [62] isotherm models were employed to give quantitative information on the adsorption of EB and TT. The nonlinear forms are presented as follows:

(6) $$Q_{eq}=KC_{eq}^{\;\left(1/n\right)}$$

(7) $$Q_{eq}=\frac{bQ_{max}C_{eq}}{1+bC_{eq}}$$

where 1/*n* is the dimensionless unit expressing surface heterogeneity and *K* [(mg g^−1^) (L mg^−1^)^1/*n*^] is a Freundlich constant expressing adsorption capacity; *Q_max_* (mg g^−1^) is the Langmuir constant, which gives the maximum adsorption capacity of the biochar with monolayer surface coverage, and *b* (L mg^−1^) is constant related to the affinity of biochar binding sites. 

**Figure 9:**
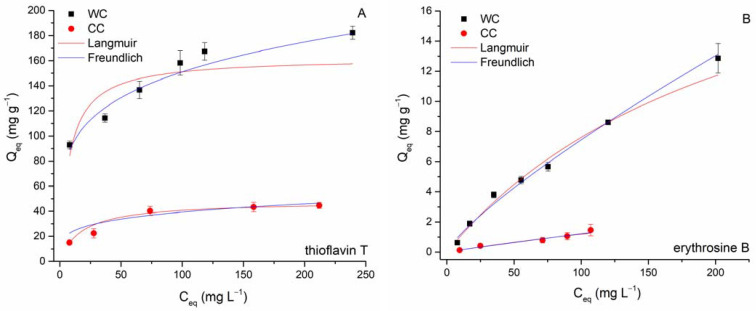
Langmuir and Freundlich isotherms for thioflavin T (**A**) and erythrosine B (**B**) adsorption by wood-chip and corn-cob biochars (8.3 g L^−1^; T = 22 °C; pH 6.0).

Figure 9 shows the results of the nonlinear fitting of the Freundlich and Langmuir models, and Table 4 presents the obtained isotherm parameters for the separation of EB and TT by biochars. Adsorption of both TT and EB by wood-chip biochar has a Freundlich isotherm shape (the slope decreases with increasing dye concentration), whereas adsorption of TT by biochar from corn cobs is rather characterized by a Langmuir-type isotherm (saturation curve shape). The observed variations are presumably due to different properties (e.g., SSA), structures of the biochars studied (see discussion above), and the different affinities of the investigated biochars for the dye molecules. Greater *R*^2^ values for the Freundlich isotherm suggest that the adsorption of TT and EB on WC can be ascribed to Freundlich surface adsorption mechanisms (i.e., indicating biochar surface heterogeneity). Both values of the exponent 1/*n* were less than 1, causing the isotherm curve to decrease at higher concentrations of EB and TT because the accessible sorption sites on the biochar outer and inner surfaces were occupied and fewer EB and TT molecules were adsorbed from the solution (Figure 9). The maximum adsorption capacities (*Q_max_*) at 22 °C calculated from the Langmuir model for TT were 162 mg g^−1^ (WC) and 48 mg g^−1^ (CC), respectively. In the case of EB sorption, it must be emphasized that the calculated *Q_max_* values (25.2 mg g^−1^ for WC and 7.5 mg g^−1^ for CC) were significantly overestimated compared to the experimentally determined maximum capacities *Q_maxexp_* (12.9 mg g^−1^ for WC and 1.6 mg g^−1^ for CC). Reflecting values of affinity constant *b*, both WC and CC biochars exhibited considerably higher affinity for TT (0.134 and 0.056 L mg^−1^) compared to EB (0.004 and 0.002 L mg^−1^).

We must note that there are only limited data for EB and TT adsorption by biochar-based adsorbents. However, comparing to other reported adsorbents of inorganic and organic origin (Table 5), the adsorption capacity of cationic dye TT onto wood-chip biochar showed a better performance. Conversely, both biochars have significantly lower adsorption capacity for anionic xanthene dye EB compared to activated carbon. The results obtained suggest that studied biochars will be more effective in separating cationic than anionic dyes from contaminated environments.

### 3.3. Impact of Biochar Characteristics on Dye Removal 

**Figure 10:**
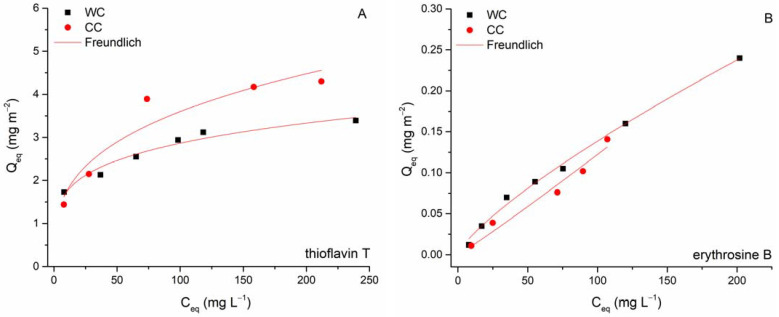
Surface-area-normalized adsorption isotherm for TT and EB on WC (**A**) and CC (**B**) biochars.

Several potential interactions for organic pollutants’ adsorption by biochar-based adsorbent have been recently proposed (see e.g., review by Tong et al. [55]). As indicated above, electrostatic attraction will not play a decisive role in the adsorption of TT by WC and CC biochars. Examined TT and EB vary significantly in molecular size (Table 1), and therefore, size-dependent adsorption on WC and CC biochars could occur, controlled by the biochar’s porosity. As mentioned above, micropores represent only 0.00005 cm^3^ g^−1^, and most of the volume of the CC was due to mesopores (0.019 cm^3^ g^−1^). Conversely, WC biochar is characterized by a uniform representation of micro- (0.021 cm^3^ g^−1^) and mesopores (0.021 cm^3^ g^−1^). Comparison of the original adsorption isotherms (Figure 9) and the surface-area-normalized adsorption isotherms (Figure 10A,B) showed that the distinctions in the adsorption affinities between the WC and CC biochars for both TT and EB are substantially larger in the original isotherms than in the case of surface-area-normalized isotherms. This suggests that a larger specific surface area and larger volume of micro- and mesopores may also contribute to the higher affinity of WC biochar to TT and indicate that pore filling represents another possible sorption mechanism for studied dyes. Moreover, the large molecular size of EB does not favor its adsorption to micropores of WC biochar due to the steric hindrance that influences diffusion into the deep tunnels and partly explains the low adsorption capacities of both biochars. 

We also tried to apply an innovative dynamic approach based on FFC NMR relaxometry to understand the nature of the adsorbate–biochar interface interactions [20]. The sorption process of thioflavin T and erythrosine B by WC and CC fully depends on water-mediated interactions. As we discussed in more detail, water dynamics differ in the porous structures of both the WC and CC biochars. Distinct relaxation times (Figure 6A,B) reveal the strength of interactions of water molecules with the pore surface of the biochar as well as with pore-water-dissolved compounds. Significantly lower relaxation times of WC indicate the presence of non-homogeneously distributed H-bond donor/acceptor groups [65]. Water is, therefore, better anchored to the WC surface compared to the CC biochar and is therefore less mobile, which may explain the better retention of both water-dissolved dyes (located and bound strongly in WC micropores). Conversely, CC with dominant macro- and mesopores and a negligible volume of micropores represents more permeable material with fast intraparticular movement of water molecules and dissolved dyes. The aqueous solution containing EB and TT, respectively, interacts with the CC biochar sorbent weaker than in the case of the WC sorbent. Moreover, part of the aqueous phase based on the notable hydrophobicity of the CC biochar remains free of contact with the surfaces of the sorbent. This suggests that part of the sorbates is out of available sorption centers. 

The presence of crystallite phases such as sylvite, quartz, and calcite (confirmed by XRD analysis, Figure 3) may also influence the overall adsorption of TT and EB. As was pointed out in recent papers, shell biochars rich in minerals exhibited high dye adsorption capacities [66,67], indicating that the mineral phases on biochar surfaces have a potential synergistic effect on dye adsorption. However, the mechanism of enhanced sorption of dyes by mineral-rich biochars is still unclear. We hypothesize that TT will be able to form complexes not only with the electron-rich aromatic structures of biochar but also with the hydroxyl moieties of inorganic silicates and other minerals present on biochar surfaces (Figure 1B). In addition to the mechanical binding of TT and EB molecules in the biochar’s pore structure, we consider the π-π interaction between biochar and sorbates to be another relevant adsorption mechanism. XRD diffractograms (Figure 3A,B) indicate the presence of aromatic carbon and the formation of turbostratic crystallites in biochar samples. The conjugated ring system acts as an π-electron donor, which can pair electron-withdrawing substituents of organics with electron-poor ring systems (e.g., benzene rings or heteroaromatic rings) [55]. Moreover, the abundant functionalization of erythrosine B by various potential hydrogen-bond donors and acceptors creates a prerequisite for additional stabilization of the associated molecules. Similarly, π-π stacking between aromatic rings of TT and the aromatic sheet structure of WC and CC biochars is expected. The presence of hydroxyl groups on both WC and CC biochars (see FTIR spectra on Figure 4 and XPS spectra on Figure 5) allows hydrogen-bond formation between H-acceptor atoms (nitrogen and oxygen) in TT and EB, and H-donors (hydroxyl groups) on biochars. 

## 4. Conclusions

The adsorption properties of TT and EB on the biochars prepared by slow pyrolysis of wood chips (WC) and corn cobs (CC) were successfully investigated and compared. The kinetics studies revealed that adsorption on external surfaces was the rate-limiting step for the removal of TT on both WC and CC biochar, while intraparticle diffusion was the rate-limiting step for the adsorption of EB. Maximal adsorption capacities *Q_maxexp_* of TT reached 182 ± 5 (WC) and 44.6 ± 2.4 mg g^−1^ (CC), and EB 12.7 ± 0.9 (WC) and 1.5 ± 0.4 mg g^−1^ (CC), respectively indicating a higher affinity of biochars for TT. The pH_pzc_ values of the WC and CC biochars obtained by the pH drift method were 8.3 and 8.5, respectively, and adsorption of both TT and EB was only slightly affected by the solution pH. The use of an innovative dynamic approach based on fast-cyclic NMR relaxometry suggests that substantial differences in the retention of water-soluble dyes can be explained by different water dynamics in the porous structures of WC and CC. Moreover, considering biochar characteristics, the π-π interaction, hydrogen bonding, pore filling, and interactions with mineral components were proposed to contribute to TT and EB removal by both adsorbents. In summary, the obtained results suggest that studied biochars will be more effective in separating cationic than anionic dyes from contaminated effluents.

## Figures and Tables

**Table 1:** Selected physicochemical properties and molecular structure of TT and EB.

Dye	Thioflavin T	Erythrosine B
Structure and size	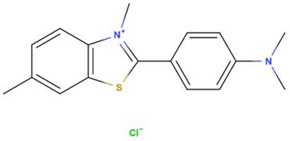 1.3 nm × 0.4 nm (l × w) [35]	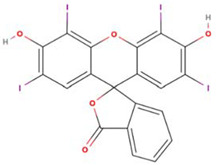 1.1 nm × 1.1 nm (l × w)
Color index number	49,005	45,430
Formula	C_17_H_19_ClN_2_S	C_20_H_6_I_4_Na_2_O_5_
MW	318.9	879.9
pK_a_	-	4.1
WS_exp_ (mg mL^−1^)	5	0.7
λ_max_ (nm)	413	526

**Table 2:** Physicochemical properties of wood-chip (WC) and corn-cob (CC) pyrolysis products.

	CC	WC
pH (H_2_O)	8.74 ± 0.07	8.58 ± 0.01
EC (mS cm^−1^)	2.39 ± 0.01	0.36 ± 0.01
ash (%)	5.65 ± 0.35	6.61 ± 0.46
Density (kg L^−1^)	0.32	0.36
C %	82.8	79.9
H %	2.08	1.59
N %	1.39	0.45
O %	9.28	11.72
S %	0.19	0.18
H/C_org_	0.31	0.24
O/C_org_	0.087	0.111
CaCO_3_ (%)	2.82 ± 0.05	0.65 ± 0.02
SSA (m^2^ g^−1^)	10.38	53.68
V_por_ (cm^3^ g^−1^)	0.021	0.045
V_micro_ (cm^3^ g^−1^)	0.00005	0.021
V_meso_ (cm^3^ g^−1^)	0.01995	0.021

**Table 3:** Kinetic parameters of thioflavin T (C_0_ = 100 mg g^−1^) and erythrosine B (C_0_ = 43 mg g^−1^) adsorption for WC and CC biochars calculated from pseudo-second-order (PSO) and intraparticle diffusion (IDM) models using nonlinear regression analysis.

Synthetic Dye	Biochar	Model	Parameter 1	Parameter 2	*R* ^2^
EB	WC	PSO	*Q_e_* = 7.41 ± 1.72	*k_2_* = 0.00006 ± 0.00003	0.971
	CC	PSO	*Q_e_* = 0.45 ± 0.004	*k_2_* = 0.0014 ± 0.0001	0.995
	WC	IDM	*k*_i1_ = 0.56 ± 0.04	*I*_1_ = −0.17 ± 0.04	0.994
			*k*_i2_ = 0.27 ± 0.08	*I*_2_ = 1.55 ± 0.28	0.827
	CC	IDM	*k*_i1_ = 0.09 ± 0.02	*I*_1_ = −0.005 ± 0.001	0.942
			*k*_i2_ = 0.025 ± 0.007	*I*_2_ = 0.27 ± 0.04	0.911
TT	WC	PSO	*Q_e_* = 100.3 ± 2.8	*k_2_* = 0.00011 ± 0.00002	0.993
	CC	PSO	*Q_e_* = 38.7 ± 1.6	*k_2_* = 0.00034 ± 0.00006	0.987
	WC	IDM	*k*_i1_ = 18.3 ± 2.5	*I*_1_ = 30.7 ± 3.70	0.945
			*k*_i2_ = 4.86 ± 1.0	*I*_2_ = 64.8 ± 6.6	0.958
	CC	IDM	*k*_i1_ = 13.7 ± 3.5	*I*_1_ = 2.12 ± 0.39	0.883
			*k*_i2_ = 2.19 ± 0.64	*I*_2_ = 24.6 ± 2.9	0.852

*Q_e_* (mg g^−1^); *k*_2_ (g/mg min); *k_i_* (mg/g min^0.5^); *I* (mg g^−1^).

**Table 4:** Parameters of Langmuir and Freundlich isotherms for thioflavin T and erythrosine B adsorption by wood-chip and corn-cob biochars calculated using non-linear-regression analysis.

Biochar		Langmuir			Freundlich		
		*Q_max_* [mg g^−1^]	*b* [L mg^−1^]	*R* ^2^	*K* [mg g^−1^ (L mg^−1^)^1/n^]	1/*n*	*R* ^2^
WC	TT	162 ± 16	0.134 ± 0.058	0.737	57.08 ± 5.04	0.21 ± 0.02	0.962
	EB	25.2 ± 5.0	0.004 ± 0.001	0.980	0.180 ± 0.043	0.81 ± 0.05	0.993
CC	TT	48.0 ± 2.7	0.056 ± 0.008	0.981	14.2 ± 0.15	0.22 ± 0.01	0.924
	EB	7.5 ± 5.0	0.002 ± 0.001	0.969	0.016 ± 0.005	0.94 ± 0.08	0.958

**Table 5:** The maximal adsorption capacities *Q_max_* for EB and TT by various adsorbents calculated using Langmuir isotherm model.

Sorbent	*Q_max EB_* (mg g^−1^)	*Q_max_*_TT_ (mg g^−1^)	Concentration Range (mg L^−1^)	SSA (m^2^ g^−1^)	pH	T (°C)	References
Moss *Vesicularia dubyana*	-	119	20–200	-	6.0	25	[6]
Ultrathin-shell BN hollow spheres	-	153	- **	175	-	-	[18]
Hop leaf biomass	-	77.48	40–400	-	6.0	25	[63]
Montmorillonite (modified)	-	95.0	- **	-	6.0	25	[17]
* Fomitopsis carnea *	-	21.9	100 ***	-	-	30	[64]
Magnetically functionalized *R. squarrosus*		154	50–400	-	6.0	22	[12]
Wood-chip biochar	12.9 *	162	13–309 (EB) 25–400 (TT)	53.7	6.0	22	This work
Corn-cob biochar	1.6 *	48	13–309 (EB) 25–400 (TT)	10.4	6.0	22	This work
Pumpkin-seed hulls	16.4	-	100–400	-	5.6	25	[54]
lemon-peel activated carbon	296	-	- **	-	4.0	25	[14]
Commercial activated carbon	77.2	-	50–450	820	7.0	20	[13]

* experimentally determined; ** not specified in original article; *** varying adsorbent dose in fixed initial concentration of adsorbate.
